# Deep learning in chromatin organization: from super-resolution microscopy to clinical applications

**DOI:** 10.1007/s00018-025-05837-z

**Published:** 2025-08-29

**Authors:** Mikhail Rotkevich, Carlotta Viana, Maria Victoria Neguembor, Maria Pia Cosma

**Affiliations:** 1 Centre for Genomic Regulation (CRG), The Barcelona Institute of Science and Technology, Barcelona, 08003 Spain; 2 Universitat Pompeu Fabra (UPF), Barcelona, 08003 Spain; 3 Medical Research Institute, Guangdong Provincial People’s Hospital (Guangdong Academy of Medical Sciences), Southern Medical University, Guangzhou, 510080 China; 4 ICREA, Pg. Lluís Companys 23, Barcelona, 08010 Spain; 5 Instituto de Biologia Molecular de Barcelona (IBMB), CSIC, Barcelona, 08028 Spain

**Keywords:** Chromatin structure, Artificial intelligence (AI), Nuclear organization, Single molecule localization microscopy (SMLM), Super-resolution microscopy (SRM), Convolutional neural networks (CNNs), Transformer architectures, Image segmentation, Single-particle tracking (SPT), STORM, Live-cell analysis, Fluorescence imaging, Molecular diagnostics, Image restoration

## Abstract

The 3D organization of the genome plays a critical role in regulating gene expression, maintaining cellular identity, and mediating responses to environmental cues. Advances in super-resolution microscopy and genomic technologies have enabled unprecedented insights into chromatin architecture at nanoscale resolution. However, the complexity and volume of data generated by these techniques necessitate innovative computational strategies for effective analysis and interpretation. In this review, we explore the transformative role of deep learning in the analysis of 3D genome organization, highlighting how deep learning models are being leveraged to enhance image reconstruction, segmentation, and dynamic tracking in chromatin research. We provide an overview of deep learning-enhanced methodologies that significantly improve spatial and temporal resolution of images, with a special focus on single-molecule localization microscopy. Furthermore, we discuss deep learning’s contribution to segmentation accuracy, and its application in single-particle tracking for dissecting chromatin dynamics at the single-cell level. These advances are complemented by frameworks that enable multimodal integration and interpretability, pushing the boundaries of chromatin biology into clinical diagnostics and personalized medicine. Finally, we discuss emerging clinical applications where deep learning models, based on chromatin imaging, aid in disease stratification, drug response prediction, and early cancer detection. We also address the challenges of data sparsity, model interpretability and propose future directions to decode genome function with higher precision and impact.

## Introduction

**Fig. 1:**
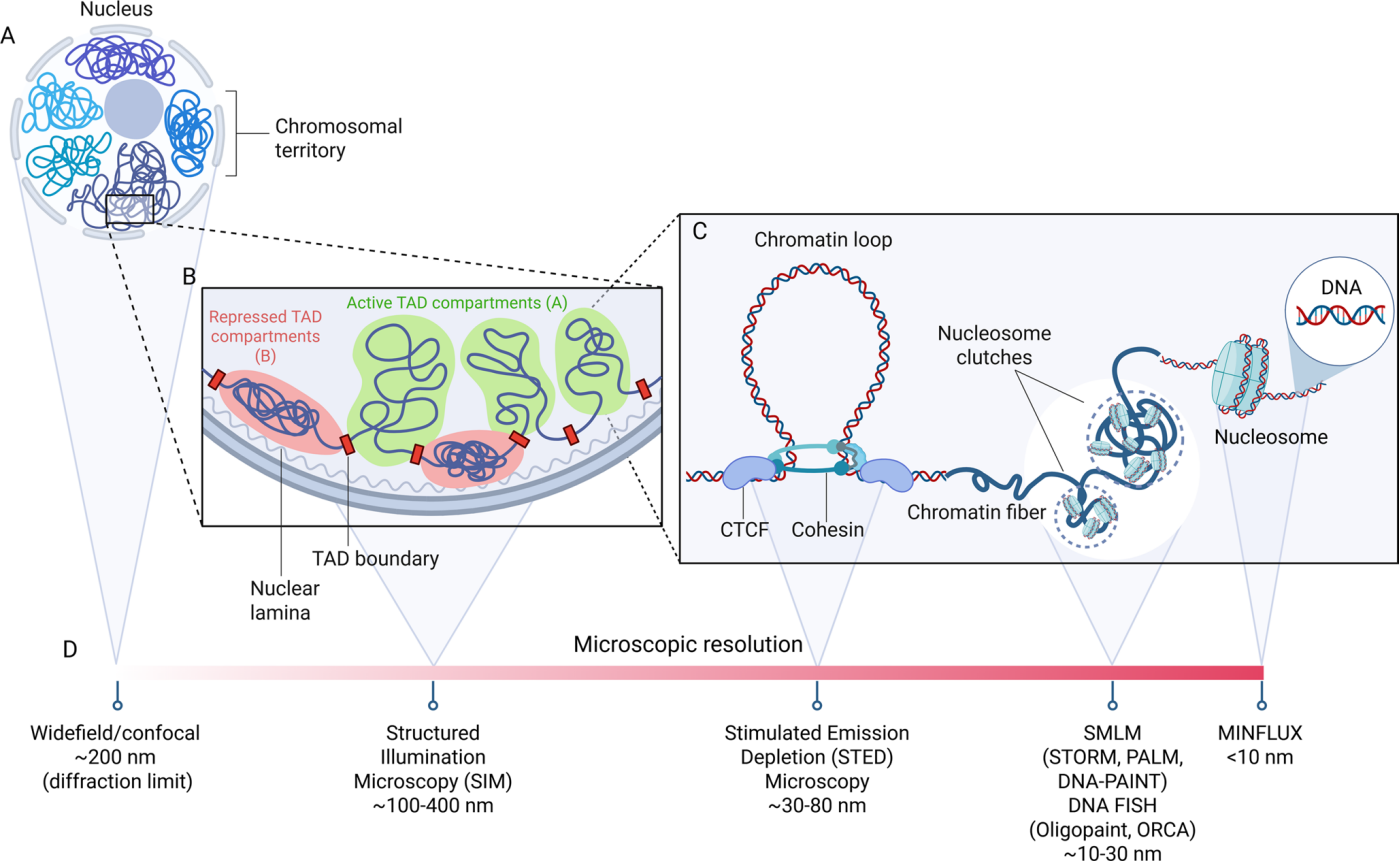
Schematic representation of the 3D genome organization and the microscopy techniques available to study it. (**A**) Chromosomes occupy discrete areas of the nucleus called chromosomal territories. (**B**) Inside each chromosome, transcriptionally active A compartments (in green) segregate from inactive B compartments (in red). (**C**) Compartments are organized into big TADs (∼Mb) and smaller loops (∼kb). Loops are formed by the chromatin extrusion through the cohesin ring, which finally stalls at CTCF anchoring points. The loop is constituted by the chromatin fiber, which contains heterogeneous groups of nucleosome clutches interspersed along the genome (highlighted in dashed circles). Nucleosomes are formed by a histone core around which the DNA helix is wrapped. Overview of available microscopic techniques, their respective spatial resolution and the associated smallest (**D**) nuclear structure that could be potentially visualized with each

Inside the nucleus, chromatin is organized in a coordinated and hierarchical three-dimensional manner [1, 2]. During interphase, chromosomes occupy specific areas of the nucleus named chromosome territories (Fig. 1A) [2]. Within these territories, chromatin is mainly organized into two compartments, called A and B (Fig. 1B) [1, 2]. More actively transcribed chromatin is mainly found in the A compartment, associated with active histone modifications, while regions of less transcription and repressive histone marks associate with the B compartment. Within compartments, chromatin is organized into Topologically Associated Domains (TADs) and loops, which are formed by the arrangement of chromatin into CTCFs-flanked loops formed by cohesin-mediated loop extrusion (Fig. 1C). At the smallest scale, chromatin is formed by repeating units called nucleosomes, formed by 146 base pairs of DNA wrapped around octamers of the four core histone proteins (H2A, H2B, H3, and H4) [3]. Nucleosomes are then arranged into nucleosome clutches, which are heterogeneous in size and density [4, 5]. Bigger and denser clutches associate with heterochromatin, and are characterized by a greater presence of H1 histone, while smaller and low-density clutches co-localize with RNA Polymerase II, suggesting a more active transcription state. Clutch size and density are also found to inversely correlate with cell pluripotency, suggesting that clutch organization is cell type specific [4]. This complex self-organization is, in turn, heterogeneous across cells and tissues and influences many processes of evolution, genome conservation, and diseases [6]. During different cellular processes, such as proliferation and differentiation, nuclear remodeling helps reshape the chromatin landscape, facilitating or restricting access to the genetic information encoded in DNA. Understanding the link between genome organization and transcription, and its functional implications, would help us understand key cellular functions and as a consequence the nature of diseases caused by aberrant chromatin folding and subsequent altered gene activity.

The knowledge about genome architecture has notably advanced in the past decades thanks to the development of multiple techniques and the declining cost of sequencing and nucleotide synthesis, allowing for complex barcoding strategies and single cell applications (Pedrotti et al., 2024) [7]. Among sequencing-based approaches, technologies like high-throughput chromosome conformation capture (Hi-C), genome architecture matrix (GAM), Micro-C and Split-Pool Recognition of Interactions by Tag Extension (SPRITE), among others, have been instrumental in revealing the hierarchical organization of chromatin at multiple scales [8–13]. Apart from single-cell Hi-C, which still faces significant technical hurdles, all these methods provide only an average view over millions of cells, underestimating single-cell heterogeneity and rare cell populations [14, 15]. The development of super-resolution (SR) microscopy strategies allowed for mapping the 3D genome with high spatial resolution at the single-cell level and opened new possibilities for studying chromatin-protein interactions. Compared to diffraction-limited microscopy, which cannot resolve structures closer than ~ 200 nm apart, SR approaches allowed the study of global genome organization with a 1–200 nm resolution (Fig. 1D) [5, 16–19]. These include structured illumination microscopy (SIM), single-molecule localization microscopy (SMLM, including STORM, PALM, and DNA-PAINT), MINimal photon FLUXes (MINFLUX) [19] and stimulated emission depletion (STED) microscopy. Thanks to the development of high-throughput multiplexed fluorescent in situ hybridization (FISH) methods, many DNA loci can be visualized across numerous single cells through sequential rounds of hybridization of oligonucleotides and imaging via microfluidic systems [17]. Examples are Oligopaints, which use custom-designed oligonucleotide probes to visualize specific DNA loci in cells [20], and Optical Reconstruction of Chromatin Architecture (ORCA), that combines the Oligopaints technology with high-resolution imaging to trace the three-dimensional organization of chromatin at single-cell resolution [21]. Moreover, the study of 3D genome dynamics has become possible by combining live-cell imaging and single-particle tracking (SPT) techniques, which allows tracing the motion of individual molecules or chromatin loci [22]. Lastly, modeling studies have recently helped in deciphering physical principles controlling genome organization and assisting with the interpretation of imaging datasets [16].

However, complexity and the volume of data generated by these techniques keep growing, making data processing and interpretation increasingly challenging. The incorporation of artificial intelligence (AI) approaches improved data acquisition, data analysis and functional prediction, overall increasing throughput and quality. Consequently, recent years have seen a surge in the application of AI, particularly deep learning (DL), in biological image and data analysis. These methods enable automated interpretation of large datasets by learning feature representations directly from raw inputs, outperforming traditional image analysis approaches in tasks ranging from segmentation to phenotype prediction. In chromatin biology, DL models have helped reconstruct 3D genome structures [23], predict cell states from nuclear morphology [24–29], and classify subcellular localization patterns with high accuracy and scalability [30].

This review focuses on the intersection of chromatin architecture and AI, particularly DL, examining how DL-based image analysis, data integration, and modeling techniques are advancing our understanding of nuclear structure at nanoscale resolution. We highlight recent breakthroughs, including the use of convolutional neural networks (CNNs), graph neural networks (GNNs), generative adversarial networks (GAN), recurrent architectures, and denoising autoencoders in tasks such as 3D reconstruction, segmentation, and dynamics tracking. We also discuss the biological insights these approaches enable such as the identification of functional chromatin states and the prediction of transcriptional outcomes, as well as the current limitations and future directions of this rapidly evolving field.

## Overcoming limitations in SMLM image reconstruction

Single Molecule Localization Microscopy (SMLM) enables imaging beyond the diffraction limit by precisely localizing individual fluorescent molecules. The fundamental principle of SMLM is the temporal separation of fluorescence emission, achieved by detecting only a sparse subset of fluorophores within a densely labeled sample at any given time. This sparsity is accomplished through various mechanisms: stochastic photoswitching (as in STORM), photoactivation (as in PALM), sparse labeling (used in Single Particle Tracking, SPT [22]), or transient binding of fluorophores to their targets (as in DNA-PAINT [31]).

A crucial component in SMLM is the point spread function (PSF), which describes how a microscope blurs a point source of light and defines the microscope’s resolution [32]. It is essential for image formation, deconvolution, and SR imaging techniques as it prevents signal overlap from multiple fluorophores within the diffraction-limited area. The controlled activation or blinking of fluorophores ensures that, in each frame, only a limited number of spatially separated PSFs are detected, allowing precise localization of individual molecules [33, 34]. The super-resolved image is reconstructed from thousands of frames in a time-lapse movie. In each frame, the positions of the active fluorophores are determined by identifying the center of their PSFs. These spots are then mathematically modeled using a two-dimensional Gaussian function, allowing for highly accurate localization of each molecule. Over time, all these localizations are combined, and any drift in the sample or microscope stage is corrected.

Precise and efficient image reconstruction is critical, as it enables accurate measurements of molecular distributions, interactions, and dynamics. However, the reliance on high photon counts for precise localization creates sensitivity to background noise and optical aberrations, while the stochastic nature of fluorophore blinking introduces artifacts such as pseudocluster formation [35, 36]. Moreover, the temporal resolution is constrained by the speed at which fluorophores can be localized which depends on the photoswitching, photoactivation or binding kinetics. As a result, acquiring complete reconstructions often requires long acquisition times. These restrictions have prompted the integration of DL approaches, which improve upon conventional techniques by learning to denoise, deconvolve, and even predict molecular localizations from raw, overcrowded, or undersampled data. Unlike classical algorithms, DL models can generalize across varying imaging conditions, compensate for experimental imperfections, and significantly speed up reconstruction without sacrificing accuracy, thus improving both spatial and temporal resolution.

The review written by Hyun and Kim provides an extensive review and comparison of most DL methods developed up to 2022 that focus on addressing the limitations of super-resolution imaging [37]. Here we focus on some of them, which have been vastly used in biomedical research, as well as on later methods, interesting for chromatin studies (Table 1).

**Table 1:** Summary of DL approaches to improve SMLM image reconstruction (classified by application, and ordered by year of publication)

Goal	Method	Architecture	Improvements	Ref.
Denoising, deblurring and improved speed	**Deep-STORM**	Encoder-decoder U-shape	SR image reconstruction from raw data; reduced data-processing time and parameter tuning	[38]
	**ANNA-PALM**	U-Net, GAN	Image reconstruction from low-density images; reduced acquisition time	[39]
	**Deep-PALM**	Encoder-decoder U-shape	Deep-STORM applied to PALM	[40]
	**U-PAINT**	U-Net	Image reconstruction with only 10% of the original DNA-PAINT raw data	[41]
	**FID-STORM**	9-layer network organized into Input Blocks, ResBlocks and DeconvBlocks	Faster version of Deep-STORM	[42]
	**DsSMLM**	Two-network-based DL framwork: U-net + DeepCNN	Localization extraction from low-photon budgeted spatial images. Improved spectral peak detection	[43]
Denoising	**Noise2Void**	U-Net based	Denoising without traditional training data	[44]
	**CARE**	U-Net based		[45]
3D imaging	**smNet**	ResNet-like CNN	Improvement of 3D coordinates of PSFs	[46]
	**DeepLOCO**	CNN with FC layers with Residual Connection		[47]
	**Deep-STORM3D**	CNN with skipped connections		[48]
	**DECODE**	Two stacked U-Nets19 with identical layouts		[49]
	**easyZloc**	MobileNet V3	Reconstruction of 3D SR images starting with 2D SMLM	
Single conventional image	**Wang** et al., **2019**	U-Net, GAN	SR image reconstruction from diffraction limited image	[50]
	**Single-frame SR microscopy (SFSRM)**	Signal Enhancement Network (SEN) and Super-Resolution Network (SRN) (enhanced ESRGAN structure– U-Net)		[51]
	**X-Microscopy**	UR-Net-8 and X-Net		[52]
	ESRGAN implementation	ESRGAN structure		[53]

### Image reconstruction for super-resolution microscopy

In SMLM, raw image frames are noisy and blurred by optical diffraction. Traditional signal processing methods rely on approaches that exploit known noise and blur characteristics. Denoising focuses on isolating individual blinking events from high levels of background and photon shot noise. Simple denoising filters such as Gaussian smoothing, mean/median filters and wavelet-based thresholding can remove noise at the cost of resolution [54, 55]. Block-matching and 3D collaborative filtering (BM3D), which denoises by jointly filtering groups of similar image patches, was adapted for high-resolution electron and single-molecule images, achieving substantial improvements in localization precision under Poisson noise [56].

Deblurring is typically framed as the inversion of the imaging point PSF. Wiener filtering performs frequency-domain deconvolution under additive noise assumptions, balancing resolution gain against noise amplification, and has long been the standard reconstruction step in structured illumination microscopy, for instance [55, 57]. Iterative approaches such as Richardson–Lucy deconvolution apply the expectation–maximization algorithm to maximize the Poisson likelihood of the blurred image given a known PSF [58, 59]. While effective, these classical methods rely heavily on accurate PSF modeling and manual parameter tuning.

**Fig. 2:**
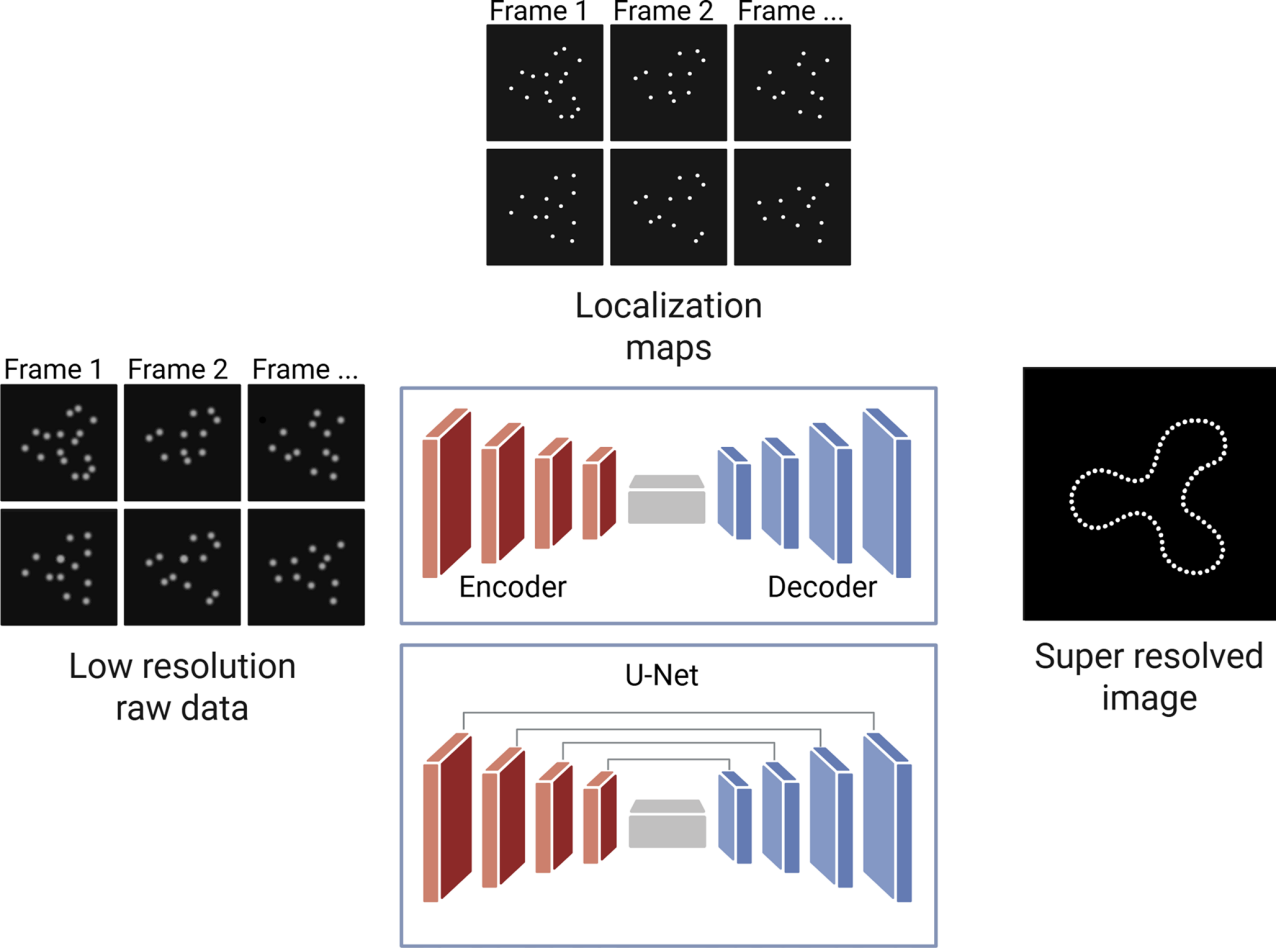
Representation of image reconstruction with deep learning. The raw low-resolution images are processed by either an encoder-decoder model, which reconstructs images via a bottleneck, or by a U-Net, which adds skip connections to better preserve spatial details. Both approaches transform low-resolution inputs into super-resolved outputs

In contrast, deep learning methods learn content-aware priors and noise characteristics directly from data, enabling robust and generalized image restoration. Introduced in 2018, **Deep-STORM** paved the way for future DL applications [38]. It is based on a fully convolutional network composed of repeated convolution, pooling, and upsampling operations that follows a U-shaped encoder-decoder design (Fig. 2). This method enables rapid, ultra-precise super-resolution reconstructions from raw blinking data, accelerates data processing and improves the reconstruction of noisy and dense datasets. It does not require specialist expertise; it is parameter-free and is easily adaptable to any single-molecule dataset.

Similarly, **ANNA-PALM** (Artificial Neural Network Accelerated PALM) employs a generator built on the U-Net architecture, combining a contracting path and an expansive path with concatenated feature maps to reconstruct high-density SR images from sparse localization and widefield inputs [39]. Specifically, the U-Net is a U-shaped CNN architecture composed of an encoder (contracting path) and decoder (expansive path) that are connected by symmetric skip connections that concatenate feature maps at each resolution (Fig. 2). Critically, its symmetric skip connections merge low-level spatial details (essential for denoising) with high-level context (vital for deblurring), effectively automating and generalizing classical restoration principles within a single end-to-end trainable framework. This method is employed to reconstruct high-density SR 2D images from sparse localization microscopy data and widefield images, reducing the time of acquisition and the number of frames needed to have a good SR image. Recently, using both experimental and simulated single-molecule images of λ-DNA, ANNA-PALM was applied to reconstruct continuous DNA contours and directly visualize nanoscopic entanglement dynamics, revealing heterogeneously distributed localization densities and the stochastic, subdiffusive migration of entanglement loci [60].

The innovation of Deep-STORM and ANNA-PALM made it possible, in the following years, for the development of faster and more advanced innovative DL methods. An example is **Deep-PALM**, an approach developed in 2020, combining Deep-STORM with PALM to study the relationship between chromatin structure and dynamics in live SR imaging [40]. Deep-PALM, trained on simulated images of three fluorophore densities, enables temporal resolution of live cell SR images of histone H2B, showing dense accumulations of H2B signal (called by the authors “chromatin blobs”). Moreover, the combination with a computational chromatin modeling tool showed the dynamic assembly and disassembly of the blobs, which likely correspond to sub-TADs, within less than one second, suggesting a close relationship between instant chromatin density and future chromatin dynamics, and vice versa.

A few years later, **U-PAINT** was developed to improve the reconstruction of DNA-PAINT images [41]. DNA-PAINT technique provides high spatial resolution, but it generally requires tens of thousands of frames and long exposure times to reconstruct a single image, which limits its applicability and throughput. U-PAINT was proven to reconstruct high-quality SR images using only 10% of the original DNA-PAINT raw data (1000–3000 frames), reducing imaging time from 50 min to just 2–5 min. Thus, this method boosts the performance of DNA-PAINT, opening new possibilities in the field of 3D genome organization by accelerating multimolecular imaging.

In 2023, a faster version of Deep-STORM, called **FID-STORM** (Fast dense Image reconstruction based Deep learning in STORM), was designed using a residual network directly on low-resolution raw images, avoiding heavy upsampling computations [42]. This method, tested on microtubules, achieved a speed of 7.31 ms/frame at 256 × 256 pixels, enabling real-time imaging, resulting in ~ 26× speed gain over Deep-STORM without sacrificing image quality. Though chromatin-specific experiments are not directly shown, the method’s ability to resolve dense structures at high speed makes it applicable to chromatin imaging. Moreover, an ImageJ plugin for FID-STORM has been developed, making it accessible to researchers in cell biology and related fields.

Critical biological events may be missed if they occur between frames, particularly when acquisition speed is slower than the dynamics of interest. DL offers a temporal SR to fill gaps. **DBlink** enables high-speed super-resolution of dynamic SMLM data, capturing long-range temporal dependencies [61]. This results in a super-resolved video reconstruction at a much higher frame rate. In practice, raw SMLM frames are first processed (e.g., by Deep-STORM) into super-resolved localization maps per frame, which are then processed sequentially by the Bidirectional Long Short-Term Memory (BiLSTM) network, which analyzes the sequence to find patterns or changes over time and interpolates missing localizations between frames. Saguy et al., demonstrated DBlink on simulations of moving filaments and mitochondria, as well as real live-cell PALM data. DBlink reconstructed dynamic processes at millisecond timescales (roughly 10× temporal upsampling) while preserving spatial resolution. Conceptually, temporal interpolation networks like DBlink can allow researchers to probe faster chromatin processes than raw frame rates usually permit. It could help in the visualization of the dynamic folding and unfolding of chromatin at millisecond timescales, could resolve fast and transient genomic interactions such as enhancer-promoter loops or phase-separated domains, and could track spatial mobility of chromatin loci in live cells with improved fidelity, aiding in the understanding of how 3D genome organization changes in response to cues like transcription or replication stress.

Finally, in 2024, a superior spatial resolution was achieved with **DsSMLM**, a deep learning-based algorithm designed for spectroscopic SMLM (sSMLM) data. This imaging method simultaneously captures a spatial image of blinking fluorophores and a corresponding spectral image of their emission, enabling more precise localization and multiplexed detection of single molecules [43]. DsSMLM leverages two neural network architectures: U-Net for the detection of blinking events in the spatial image frames, and Deep CNN (DCNN) with a skip connection to improve spectral peak detection from spectral frames. This process enhances spatial resolution and allows for multicolor imaging even with overlapping spectral features. DsSMLM, combined with a label-free imaging technique, allowed for the visualization of DNA with a spatial resolution of 6.22 nm, avoiding artifacts introduced by antibodies or dyes and revealing native chromatin features. DsSMLM has also been applied to visualize histone modifications, finding an enrichment of H3K9me3 localizations at the nuclear periphery and nucleoli, and the presence of H3K4me3 in the nuclear interior. This method achieves an 8.8% higher localization count for labeled histone markers compared to conventional sSMLM, and improves spectral peak accuracy, reducing misidentification and enhancing contrast.

Finally, only focusing on denoising, **Noise2Void** (N2V) [44] is a self-supervised CNN that uses a U-Net architecture with a “blind spot” where the central pixel of a receptive field is masked during training. This prevents the network from learning to output exactly what it gets as input, and forces it to rely on the structure of the surrounding pixels to predict the denoised value. N2V workflow has been further implemented by Hajiabadi and colleagues to study the 3D shape of RNA Polymerase II (Pol II) clusters [45]. This method allowed the observation of stereotyped, transient changes (~ 10 s) in Pol II clusters: activated Pol II intensity increases first, indicating recruitment, and then rises, suggesting transcription initiation. Moreover, the cluster shape shifts from round to elongated, possibly reflecting gene engagement and elongation.

Moreover, Kefer and colleagues applied both supervised content-aware image restoration (**CARE**) based on the U-net CNN architecture [62] and N2V denoising to fluorescence movies of photoactivated chromatin microdomain arrays (PAGFP-H2A) to improve their tracing in live cells [63]. Both CARE and N2V sharply enhanced image quality (peaks became well-defined) and dramatically increased the fraction of microdomains that could be tracked, especially in noisy short-exposure sequences. After N2V denoising, inferred diffusion coefficients from tracked microdomains closely matched the ground truth. Notably, N2V did not mask real reductions in chromatin motion (e.g., after drug treatment). However, at extreme noise (very short exposures), CNN denoising sometimes “hallucinated” spurious spots and underestimated diffusion coefficients. Thus, content-aware denoising (CARE or N2V) can recover true chromatin motion, increasing tracking accuracy over classical methods; the main caveat is careful training and validation to avoid artifacts at very low signal-to-noise ratio (SNR) images.

### Enhanced 3D imaging

DL methods have also been developed for the analysis of 3D STORM, an extension of 2D STORM which adds axial (z-axis) resolution, typically ~ 20–50 nm, allowing detailed 3D reconstructions of biological structures. DL methods such as **smNET** [46], **DeepLOCO** [47], **DECODE** [49] and **Deep-STORM3D** [48] determine the 3D coordinates of PSFs with higher accuracy than traditional Gaussian fitting approaches and thus help reconstruct a clearer 3D SR image. For instance, Deep-STORM3D has been employed to characterize telomere diffusion during interphase in live cell imaging, demonstrating cell cycle phase-dependent local chromatin constraints and the regulation of chromatin diffusion and chromosome segregation by Lamin A [64].

A recently developed approach, **easyZloc**, can reconstruct 3D SR images starting from 2D SMLM images acquired with a standard, unmodified fluorescence microscope [23]. This method uses a calibration dataset to capture a z-stack of sub-resolution fluorescent beads across the axial (Z) range of the microscope, and learns how the PSF changes with Z. On new 2D localizations, the trained network predicts the Z-position, turning each 2D point into a 3D coordinate. While the focus is broader than chromatin alone, chromatin-related structures were analyzed via easyZloc, which successfully reconstructed the dual-ring structures of nuclear pore complexes with axial precision comparable to state-of-the-art methods like the aforementioned.

### SR image reconstruction from low-resolution images

In light of all the previously discussed improvements driven by DL integration in microscopy, another promising application is image reconstruction of SR images directly from conventional diffraction-limited widefield (WF) images. This approach allows to move towards more accessible and enhanced SR imaging, producing high-resolution images without sophisticated optical setups or extensive computational post-processing.

Among the foundational efforts, in 2019, Wang and colleagues proposed an architecture based on Generative Adversarial Network (GAN) to enhance the resolution of fluorescence microscopy images across multiple modalities, without numerical modeling or point spread function estimation [41]. Their framework employs a U-Net-based **generator** with residual convolutional blocks and leaky ReLU activations to transform low-resolution inputs into super-resolved outputs through feature extraction and upsampling. The discriminator, implemented as a convolutional neural network, evaluates perceptual realism by distinguishing generated images from ground-truth high-resolution data using adversarial loss. This adversarial training regime forces the generator to preserve biological structures while suppressing artifacts, achieving cross-modality resolution enhancement (e.g., confocal → STED).

Building on this concept, methods such as Single-Frame Super-Resolution Microscopy (**SFSRM**) and **X-Microscopy** have further expanded the capabilities of DL in fluorescence imaging. In particular, **X-Microscopy** integrates two DL subnetworks—UR-Net-8 and X-Net—to reconstruct STORM-like images directly from widefield data. The system uses a mimic undersampled SRM (MU-SRM) as an intermediate step to guide the final reconstruction, enabling flexible input sizes and robust performance across a wide range of biological structures. The model was trained on simulated and experimental datasets of subcellular structures and validated with STORM images as ground truth. This method achieved ~ 30 nm spatial resolution and, in combination with expansion microscopy, it enables visualizing chromatin-related structures like nuclear pore complexes. These tools proved the reconstruction of images of histone H2B and the H3K9me3 heterochromatin marker, closely resembling full STORM images.

Similarly, Lossano et al. adapted the Enhanced Super-Resolution GAN (ESRGAN) architecture to fluorescence microscopy, using a perceptual-driven training approach to convert WF images into high-resolution outputs [53]. Trained with paired WF-STORM datasets and fine-tuned through transfer learning, this method enables rapid, high-throughput nanoscale imaging with significantly reduced acquisition time.

While GAN-based architectures offer powerful SR reconstruction capabilities, they remain sensitive to imperfections in ground truth datasets. Artifact propagation (e.g., stemming from training data with uneven blinking statistics, photobleaching effects) becomes encoded into network outputs [53, 65]. Nonetheless, the ability to generate STORM-like images from rapidly acquired widefield data opens possibilities for real-time dynamic studies, high-throughput screening applications, and multicolor imaging protocols that were previously impractical. As technology continues to mature, this approach may fundamentally transform how researchers approach nanoscale cellular imaging, making advanced super-resolution capabilities more accessible and practical in standard lab environments.

## Deep learning methods for improved SR image segmentation

The next common challenge in SR image analysis is segmentation, which is the process of identifying and classifying objects within images [66]. It is essential for quantifying cells, subcellular structures, and molecular complexes, enabling insights into biological processes like cell division, morphogenesis, and growth. There are two main segmentation approaches: semantic segmentation, which labels each pixel by class, and instance segmentation, which identifies individual objects [67]. The latter is commonly used to distinguish each cell or structure as a separate entity [68]. However, manual segmentation is slow, error-prone and biased, while automated methods face difficulties due to structural variability, image noise, overlapping objects, and the need for significant computational resources and annotated data. Recently, DL-based methods have been developed to overcome all these limitations, achieving unbiased, highly accurate automatic segmentation (Table 2).

**Table 2:** Novel segmentation approaches related to nuclear segmentation (classified by architecture, and ordered by year of publication)

Architecture	Name	Description and improvements		Ref.
U-Net	Retrained version of Cellpose	Segmentation of specific nuclear regions		[69]
	csPWS-seg	Segmentation of live cell label-free imaging data from Chromatin-sensitive partial wave spectroscopic (csPWS) microscopy. Outperformed performance of previous methods.		[70]
	aiSEGcell	Nuclear segmentation from bright filed images		[71]
R-CNN	SR trained Mask R-CNN	Improved SR image segmentation		[72]
	Enhanced Rotated Mask R-CNN	Chromosomal segmentation		[73]
GAN-based	SimOptiGAN+	3D image segmentation; trained on synthetic data to better represent rare cell states	Places real nucleus shapes inside simulated cells, then improves the image. Most accurate, great for training AI	[74]
	Mem2NucGAN-P		Learns from paired real examples (e.g. membrane + nucleus). Easier to train, no need to place nuclei manually	
	Mem2NucGAN-U		Learns from separate membrane and nucleus images. Looks the most realistic, flexible	

Biomedical image segmentation is usually conducted with U-Net or Region-based CNN (R-CNN) architectures [75–78]. **CellProfiler** [79] and **Cellpose** [28] are exemplary U-Net-based software that are widely utilized in biomedical research. Both are free and open-source, featuring user-friendly interfaces that facilitate the segmentation, measurement, and analysis of cell images. The encoder layers identify the relevant features from the input image and reduce its spatial resolution, while the decoder layers take the encoded information and use it to generate a segmentation map, locating the features without losing the spatial resolution of the input. The output is a binary segmentation map where each pixel represents a foreground or a background region. This method provides fast and precise segmentation, working well with limited training data thanks to extensive data augmentation, and can handle varying input image sizes. These methods have also been used as a basis for more complex tasks. For instance, Cellpose was retrained and adapted to segment nuclear regions from SMLM data [69]. Localizations from the membrane and the nuclear interior were analyzed separately, enabling the study of molecular patterns associated with different subcellular compartments. A recent example of a novel U-Net-based method is **csPWS-seg**, used to segment live cell label-free imaging data. This allows the recognition of specific cell structures from much more noisy images [70]. Same for **aiSEGcell**, a U-Net-based method which improved the segmentation of label-free cell nuclei from bright field images, trained only on 32 images [71].

On the other hand, R-CNN allows for the precise localization of objects within an image through the creation of bounding boxes. In brief, R-CNN starts by dividing the images into regions, called “region proposals”, which are parts of the image that are likely to contain an object. These regions are resized and passed through a CNN for feature extraction, and the extracted features through a separate machine learning (ML) classifier for each class of interest. R-CNN also performs bounding box regression to refine the location and the size of the object [76]. The original R-CNN architecture established the paradigm of combining region proposals with convolutional features but suffered from critical inefficiencies, such as computational redundancy and feature storage overhead, making large-scale SRM datasets (often exceeding 100 GB/image) impractical. Nevertheless, the R-CNN family of models has undergone significant evolution since its inception and paved the way for more efficient tools for object detection, like **Fast R-CNN**, **Faster R-CNN**, and **Mask R-CNN** [77, 80, 81]. As R-CNN-based architectures are typically trained on natural images, their application to super-resolution (SR) image segmentation faces challenges due to (1) computational inefficiency from high-resolution inputs, (2) artifacts (e.g., PSF distortions, reconstruction noise), and (3) domain shift caused by discontinuous features (abrupt changes or transitions in pixel intensity, texture, or color) and scale mismatches in SR data. Mela and Liu proposed a retraining of Mask R-CNN with SR images, drastically improving SR image segmentation [72]. A similar approach was adopted for automatic chromosome segmentation and classification with a method called **Enhanced Rotated Mask R-CNN**, which also allows the recognition of overlapping chromosomes [73].

Finally, three GAN-based methods, called **SimOptiGAN+**, **Mem2NucGAN-P** and **Mem2NucGAN-U **[74], have recently been published aiming to generate realistic synthetic nuclei images and labels for training 3D segmentation models *(*Table 2*).* These approaches address a critical bottleneck in biomedical imaging by automating the production of high-quality training data that would otherwise be labor-intensive and time-consuming to annotate manually. By incorporating biophysical constraints these GAN-based frameworks can accurately simulate complex cell arrangements and morphological variations. The resulting synthetic datasets are particularly well-suited for training deep learning models on challenging 3D structures like organoids, where manual annotation is especially difficult due to high cell density and structural complexity [82].

In conclusion, many DL tools have been developed to enable precise and rapid segmentation. The choice of tool largely depends on the specific task, the type of input images, availability of annotated data, and computational resources. These tools can often be retrained or fine-tuned to create methods tailored to specific datasets. As segmentation continues to evolve, integrating synthetic data generation and tailoring models to SR imaging will be crucial for pushing the boundaries of cellular and subcellular analysis in biomedical research.

## Deep learning approaches for enhanced single-particle tracking

AI has also transformed the ability to visualize and quantify the dynamic behavior of chromatin and associated nuclear structures. Through live-cell imaging and DL-driven analysis, researchers can now investigate chromatin mobility, nuclear body interactions, and transcriptional bursting in real time [83–86]. This section discusses the progression of methodologies for dynamic tracing and the subsequent application of ML techniques to decipher particle motion patterns, highlighting key technological breakthroughs and their implications for biological research.

Single-particle tracking (SPT) is widely used to study chromatin dynamics, as it enables the observation and analysis of the motion of individual particles, such as proteins, vesicles, viruses, or even gene loci, within live cells [86–88]. In a typical SPT experiment, fluorescently labeled proteins or chromatin loci are imaged over time, and specialized software links their positions into trajectories. These trajectories yield diffusion coefficients, binding/dwell times, and motion types (Brownian, subdiffusive, directed, etc.) that reflect the underlying chromatin environment [89–91].

Unlike bulk measurement techniques, SPT provides detailed insights into the behavior and heterogeneity of individual molecules, making it invaluable for probing dynamic biological processes at the nanoscale. It enables researchers to directly connect molecular motion with biological function, for example, how enhancer–promoter looping (a type of long-range interaction) correlates with local mobility and chromatin remodeling [92], or how phase-separated nuclear bodies affect nearby chromatin motion [93].

SPT can also directly report the mobility and confinement of individual nuclear particles, providing insight into chromatin constraints and remodeling events, such as the decompaction effects of the NuRD complex [94] or the dynamic switching of histone H2B between chromatin states [95]. For instance, SPT of labeled loci or nucleosomes has revealed that chromatin motion is highly heterogeneous and often subdiffusive on short timescales [96–98]. Recent reviews note that SPT “can provide robust measurements of chromatin-interacting proteins in vivo” and can complement genome-wide assays [99].

Classical tools such as **TrackPy** [100] and **TrackMate** [101] have long served as the foundation for SPT, providing robust pipelines based on deterministic rules. TrackPy, implemented in Python, applies the Crocker–Grier algorithm with customizable filtering parameters (e.g., particle size, brightness, memory). TrackMate, as a plugin in Fiji/ImageJ, offers GUI-driven tracking using Laplacian of Gaussian (LoG), Difference of Gaussian (DoG), or threshold-based detection, and supports linking through linear assignment and Kalman filtering. These tools remain widely used for their transparency, extensibility, and real-time feedback during experimental analysis: TrackMate can handle 2D/3D fluorescence image stacks with customizable detection and tracking options, while TrackPy integrates well with the scientific Python stack for batch processing [102]. However, these methods depend on manually tuned thresholds and heuristics, and their performance often degrades under low SNR conditions or in the presence of complex motion patterns [103].

Recent developments address challenges posed by dense particle fields and complex biological environments. **DeepTrack 2.0** advanced this shift by fully embracing DL [104]. Its modular design allows tailoring network architectures and loss functions to specific problems, exemplified by Midtvedt et al.’s three-layer CNN, which maintained sub-pixel localization accuracy under low signal-to-noise ratio (SNR) conditions, a key challenge in live-cell SR microscopy, where classical methods like Radial Center faltered. The authors report that DeepTrack solutions generalize well to varying noise and background levels. Its key advantage is flexibility; it works as a toolbox, and one can tailor the network architecture, input/output formats, and loss function to a specific problem. **SPTnet** further revolutionized trajectory reconstruction through its transformer-based architecture, processing entire video sequences in parallel to capture global spatiotemporal relationships. This end-to-end DL framework eliminates traditional processing steps like particle detection and linking, directly inferring trajectories and motion parameters (e.g., diffusion coefficients) and achieving theoretical precision limits even under low SNR conditions [105]. It can resolve spatially overlapping particles across frames and generalize effectively to experimental datasets from various imaging modalities, including fluorescence and super-resolution live-cell microscopy, highlighting its strong potential for SPT in complex biological systems.

Beyond individual trajectories, some methods map chromatin motion continuously across the nucleus. **Hi-D** (high-resolution diffusion mapping) is an approach developed for dense chromatin imaging [84]. In Hi-D, the entire nuclear image (labeled DNA or a nuclear protein) is processed with optical flow to compute local displacements at each pixel. These pixel-wise trajectories are then classified by a Bayesian inference step into diffusion models, yielding spatial maps of diffusion coefficients and modes. The output is a super-resolution “movie” of chromatin motion across the nucleus. For chromatin researchers, Hi-D provides a global view: one can see, for example, whether certain subnuclear regions (like heterochromatic foci or nuclear bodies) have distinct diffusivity. Applied to live cells revealed that chromatin dynamics form mosaic domains (0.3–3 μm) that shift with transcriptional activity, and that mobility is driven by local DNA–DNA contacts and protein binding rather than simple compaction [84]. Garate et al., (2024) applied nanoscale live-cell imaging to study how chromatin organization and transcription factor dynamics change during stem cell differentiation. They observed that exit from naïve pluripotency is accompanied by global chromatin decompaction and reduced mobility of the OCT4 transcription factor [86]. Hi-D is particularly effective for characterizing spatial heterogeneity in chromatin mobility. Still, it relies on densely labeled confocal image stacks and lacks the learning capacity to generalize beyond its model assumptions.

Once trajectories are extracted, a key question is how particles move. DL excels at classifying motion without manual feature engineering. Traditional motion analysis relied on manually engineered features like mean squared displacement (MSD) or diffusion coefficients, which struggle to capture complex, heterogeneous dynamics [106].

The desire to capture not only static classification but also dynamic transitions within trajectories led to hybrid models like the back-propagation neural network (**BPNN)**, which used a simple feedforward network trained on features derived from MSD curves to detect switches among Brownian, confined, and directed motion within each trajectory [107]. This bridged the gap between feature-based analysis and learning-based adaptability: it uses MSD features explicitly rather than raw displacements. The authors used this method to classify and segment trajectories of membrane proteins by computing a short sliding window normalized MSD for each segment and feeding this MSD curve into a simple feedforward network with one hidden layer (sigmoid activations). Nevertheless, it provided an automatic, adaptive segmentation of trajectories into diffusion regimes that aligned well with known transitions in membrane protein data and that could be useful to identify transitions in chromatin binding proteins. For instance, BPNN could potentially identify binding or unbinding events of a transcription factor from its target search phases within the same track. This hybrid strategy can segment a single trajectory into mixed modes– for example, a TF might diffuse freely for a while, then transiently pause (confined) when it binds DNA. Such change-point detection is useful in chromatin, where interactions (with polymerases, chromatin remodelers, transcription factors, etc.) alter the motion regime [95, 108].

While hybrid models like BPNN introduced the ability to detect dynamic transitions within trajectories using hand-crafted features, a series of methods were developed in the context of the Anomalous Diffusion (AnDi) Challenge, which benchmarked trajectory classification/regression tasks.

By leveraging self-supervised learning to capture more complex, non-linear dynamics, Granik and colleagues implemented a CNN to infer anomalous diffusion parameters of membrane proteins’ trajectories [109]. Their deep model classifies each entire trajectory as Brownian motion, fractional Brownian motion (fBM), or continuous-time random walk (CTRW), and simultaneously regresses the Hurst exponent (for fBM) and diffusion coefficient. The network was trained on simulated data and applied to experimental trajectories, demonstrating that many cellular processes exhibit anomalous (non-Brownian) diffusion.

In a complementary study by Kowalek et al., a CNN could classify diffusion modes slightly better than traditional feature-based methods [110], albeit with longer computation time. They found that CNNs tend to outperform when large training sets are available, though classic methods remain competitive in some borderline cases. While the above-mentioned methods were proved valuable for membrane proteins, whose movement is intrinsically restricted in the Z-dimension due to their confinement within the two-dimensional plane of the lipid bilayer, these CNNs could be even more useful in the tracking of nuclear factors that typically move within the three-dimensional environment of the nucleus, which allows for more complex and less constrained trajectories.

In 2019, Carlo Manzo introduced **AnDi-ELM**, an approach combining hand-engineered features with an **Extreme Learning Machine (ELM)** classifier [111]. Here, a simple single-layer neural network (ELM) is trained quickly on a feature vector for each trajectory. Despite its simplicity, AnDi-ELM achieved competitive performance in inferring the anomalous exponent and diffusion model, offering a fast “pre-screening” tool for large datasets. Li et al., proposed **WADNet**, a deep network that uses a modified WaveNet encoder (dilated convolutions) plus a bidirectional LSTM to analyze trajectories [112]. WADNet outperformed all other methods in the AnDi challenge’s inference (exponent) and classification tasks across dimensions. It requires no prior model assumptions, learning relevant time-series features automatically. Both WADNet and AnDi-ELM illustrate that deep/statistical models can automate anomalous diffusion analysis: WADNet for maximal precision and AnDi-ELM for fast approximate screening.

To incorporate relational and temporal information from trajectories in a fundamentally new way, **Verdier** et al., developed a **Graph Neural Network (GNN)** approach [113]. By converting each trajectory into a graph (vertices = positions, edges = time steps) and applying graph convolutions to learn latent features, the GNN learned to classify diffusion modes and estimate parameters with high accuracy even for very short tracks. Trained on simulated walks (including all AnDi models), the GNN achieved high accuracy in classifying model type and predicting anomalous diffusion exponent α. Critically, the method naturally handles trajectories of any length and was shown to retain accuracy even for very short tracks (tens of points) using relatively few learnable parameters. In 2023, Pineda et al., included a GNN in the DeepTrack2.0 model that outperformed baseline models in trajectory prediction on simulated data using Anomalous Diffusion (AnDi) datasets [114]. However, GNNs models in single-particle tracking face challenges including computational complexity, memory-intensive graph construction in dense environments (e.g., O(100k) detector hits) and generalization to noisy/mixed trajectories.

More recent advances, such as **Deep-SEES** [115] and **DeepSPT** [108], enable self-supervised analysis of complex molecular motions. Deep-SEES integrates an LSTM-VAE architecture to encode spatiotemporal trajectory features into noise-robust latent representations, revealing dynamic biological states without prior kinetic knowledge. Initially validated on nanoparticle-membrane interactions and phase-separated systems, it later showed applicability for studies of phase transitions and heterogeneity in chromatin organization. The combination of lattice light sheet microscopy and single molecule imaging revealed an inverse correlation between nucleosome mobility and chromatin density (*r* = −0.48) [116], highlighting potential for resolving transient states during phase transitions. Through H2B trajectory analysis, the framework can excel at identifying transient states during chromatin remodeling events and characterizing the dynamic behavior of individual nucleosomes within different chromatin environments [95].

DeepSPT’s modular pipeline (temporal segmentation → diffusion fingerprinting → classification) directly converts raw trajectories into behaviourally annotated data. While demonstrating 82–88% accuracy in virology applications [108, 117], its motion fingerprints can map chromatin domains using nucleosome mobility patterns. When applied to chromatin, DeepSPT achieved impressive accuracy in distinguishing different chromatin compartments based solely on their diffusional properties. For example, it successfully differentiated between early endosomal (EEA1-positive) and late endosomal (NPC1-positive) compartments with up to 82% accuracy based exclusively on movement patterns [95]. This capability is particularly valuable for distinguishing euchromatin from heterochromatin domains or identifying specialized chromatin structures like transcription factories and repair foci without requiring additional fluorescent markers. Recently, three-color SMLM reveals spatial coupling between heterochromatin, euchromatin, and transcription at phase-separated interfaces [118], where diffusion barriers restrict HP1α mobility (D = 0.10 μm²/s vs. 1.78 μm²/s in nucleoplasm) [119]. DeepSPT may classify these interface constraints as distinct sub-diffusive regimes, enabling rapid, real-time monitoring of chromatin states during DNA repair [108]. Though differing in focus—latent reconstruction vs. real-time classification, these tools synergize effectively. Deep-SEES’ denoised trajectories could feed into DeepSPT’s fingerprinting module for hierarchical chromatin analysis. Implementation challenges remain in generalizing these architectures to non-Brownian motion regimes and ultra-short trajectories.

DL has opened new frontiers in the study of chromatin dynamics, enabling high-resolution, quantitative analysis of genome organization, transcriptional regulation, and nuclear body interactions. The integration of these DL methods enables a hierarchical and multi-modal approach to chromatin dynamics but remains a challenge when it comes to capturing the spatial complexity and morphological heterogeneity of chromatin structures at nanoscale resolution.

**SEMORE** (SEgmentation and MORphological fingErprinting) addresses this gap by combining segmentation with morphological fingerprinting, offering a robust framework to dissect chromatin organization from super-resolution microscopy data [120]. By generating detailed morphological descriptors and enabling high-accuracy classification of chromatin domains, SEMORE enhances our ability to map functional chromatin states and their dynamic changes, providing a critical tool for decoding genome architecture and epigenetic regulation. As super-resolution microscopy continues to advance, tools like SEMORE will be increasingly valuable for uncovering the mysteries of chromatin organization and its role in cellular functions.

Looking ahead, several key challenges remain in the study of chromatin dynamics. These include the limited availability of annotated chromatin trajectory datasets [102], the need for robust 3D analytical adaptations [121], and difficulties in interpreting features derived from DL models [122]. Achieving a comprehensive understanding of chromatin function will require the integration of chromatin dynamics data with other modalities, such as genomic, transcriptomic, and epigenomic datasets [121].

**Fig. 3:**
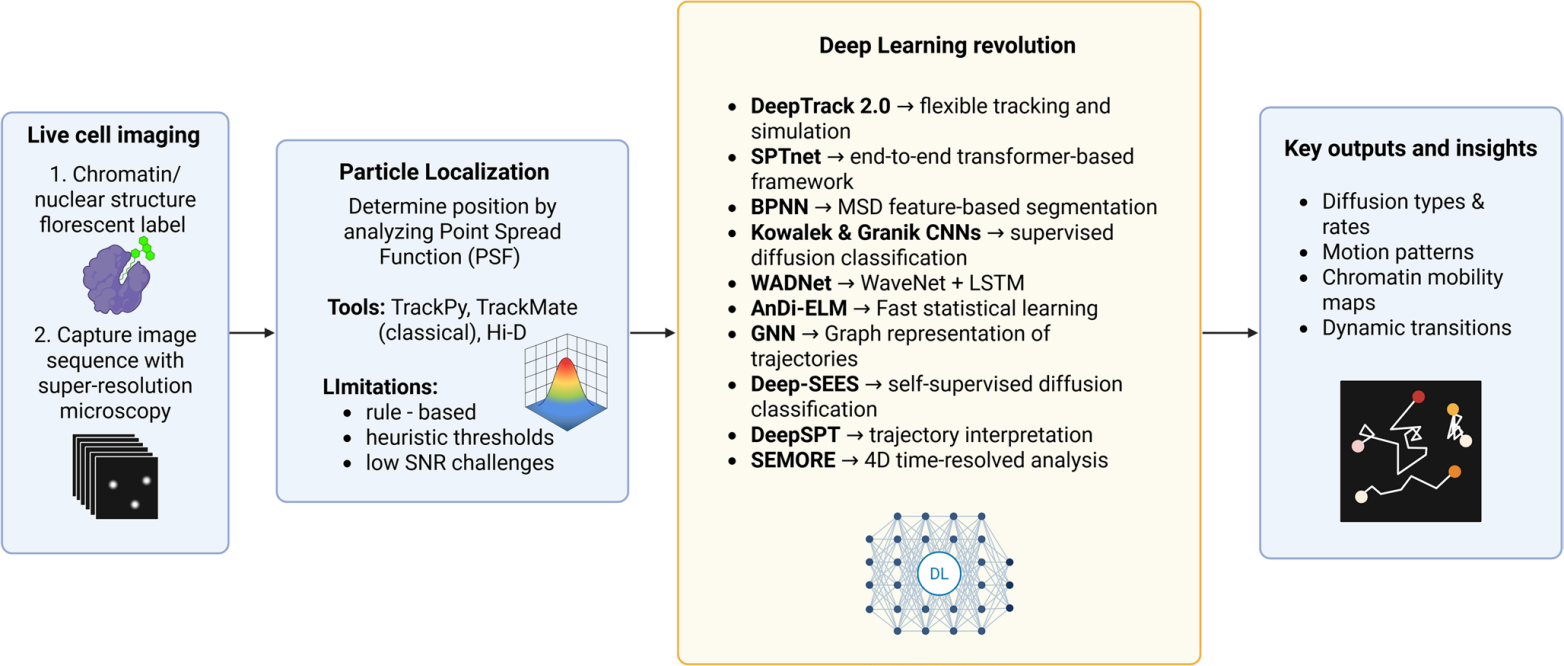
Chromatin dynamics and single-particle tracking workflow. From left to right, specific target particles (e.g. gene loci) are imaged in live cells. Images capture the motion of the particles across frames. Classical tools undergo particle localization based on the position of the point spread function and its fitting under a gaussian distribution. Novel methods (in orange box) leverage DL to improve the accuracy of particle trajectory reconstruction and get insight into the trajectory dynamics

To support broader adoption within the chromatin and nuclear dynamics community, continued development of user-friendly, open-source tools and standardized benchmarking datasets is essential. Each of the methods discussed above presents specific trade-offs. Table 3 provides a comparative summary of their model architectures, input types, target applications, performance relative to classical approaches, and reported advantages and limitations. An overview of the typical SPT analysis workflow is presented in Fig. 3.

**Table 3:** Comparison of ML methods for SPT analysis

Method	Architecture	Input Data	Application/Target	Performance	Key Advantages/Limitations	Ref.
Dosset BPNN	Feedforward net with 1 hidden layer (sigmoid neurons)	Sliding-window MSD curves (segments of trajectories)	Local diffusion mode and switching (Brownian/confined/directed)	Accurately identifies modes and transitions (down to ~ 20-frame segments)	Detects switches automatically; uses MSD features explicitly. **Limitation**: Limited to the modeled modes; window-size must be chosen; simpler than deep nets.	[107]
Granik CNN	CNN (few conv layers) with regression outputs	Short 1D SPT trajectories (both simulated & experimsental)	Classify diffusion model (BM, FBM, CTRW); infer α (for FBM) and D (for BM)	Higher accuracy and lower data requirement than MSD fitting; network required half as many trajectories to reach same precision	Effective on very short trajectories; no parameter tuning needed. **Limitation**: Architecture specialized to specific models; requires synthetic training covering parameter range.	[109]
Kowalek CNN	Deep CNN (6 conv layers + 2 FC layers)	Raw 2D single-particle trajectories (simulated)	Diffusion mode classification (Brownian/AD/directed/confined)	~ 97.3% accuracy on simulated data (slightly > classical ~ 96%). Confusion mostly on normal vs. confined.	Works on raw data without feature engineering. **Limitation**: Longer training; needs large synthetic training sets; limited by training models.	[110]
DeepTrack 2.0	User-configured CNN/U-Net/GNN/GAN models; typically several conv layers + FC	Simulated or real microscopy images (particles, cells)	Localization, tracking, counting in microscopy	Outperforms classical localization (e.g. radial center) under low SNR; high accuracy if simulations are accurate	Highly flexible, user-friendly; leverages realistic simulations. **Limitation**: Requires careful modeling of experimental noise; performance hinges on simulation fidelity.	[104, 114]
AnDi-ELM	Extreme Learning Machine (single-layer NN) with engineered features	Extracted trajectory features (e.g. TAMSD)	Infer α, classify model, segment (AnDi tasks)	“Satisfactory” accuracy on AnDi benchmark with minimal computation	Very fast to train; lightweight. **Limitation**: Simpler than top methods; relies on quality of engineered features.	[111]
WADNet	WaveNet-style dilated conv encoder + LSTM + MLP	1D trajectories (simulated AnDi data)	Infer α and classify model (AnDi tasks)	State-of-the-art on AnDi: surpassed all 6 subtasks for inference and classification	Exceptionally high accuracy on heterogeneous diffusion; captures multi-scale temporal patterns. **Limitation**: Large complex network; heavy training.	[112]
Verdier GNN	Graph neural network (node features + message-passing)	Trajectories encoded as graphs (nodes = timepoints, edges linking neighbors)	Infer model and α for anomalous diffusion (AnDi tasks)	High accuracy on AnDi walks; robust on any trajectory length, even very short (few parameters)	Naturally handles variable-length tracks; learns physics-relevant features. **Limitation**: Complex setup (graph construction); requires simulated training.	[113]
DBlink	Hybrid CNN + bidirectional LSTM network	Video of SMLM frames (or localization maps)	Dynamic super-res imaging (SMLM temporal interpolation)	Achieves ~ 10× faster SR video; outperformed previous methods on AnDi tasks	Enables millisecond super-resolution of moving structures. **Limitation**: Complex model; requires large training data from simulated/controlled motion.	[61]
Deep-SEES	Self-supervised learning using a Variational Autoencoder (VAE) framework with LSTM units.	Multivariate time series from SPT	Classify and segment dynamic states from noisy SPT data without prior labeling or diffusion models.	High accuracy in classifying different diffusion modes, Root Mean Square Error (RMSE) improved by 26% compared to baseline methods.	No prior model or labelling needed, robust to noise and undersampling. **Limitation**: dependent on rolling window size, k-means clustering constraints in latent space.	[115]
SPTnet	Transformer-based deep learning framework with 3D-ResNet backbone, two-stream decoder-encoder transformers, feature fusion module, and 4-headed headed multilayer perceptron (MLP)	Fluorescence microscopy videos (raw camera frames), single-molecule tracking photoactivated localization microscopy (sptPALM)	End-to-end single-particle tracking and motion dynamics analysis	Outperforms traditional SPT methods across a range of conditions with precision close to the theoretical limit; 27–29% better detection accuracy compare to TrackMate	End-to-end, parameter-free tracking and analysis– High precision even in low SNR, heterogeneous backgrounds, and motion blur, fast inference (~ 60 ms/video). Robust across multiple imaging. **Limitation**: Requires ~ 100 GPU hours (RTX 3080) for training	[105]
DeepSPT	Three core modules: M1: Temporal Behavior Segmentation, M2: Diffusional Fingerprinting, M3: Task-Specific Classification	2D or 3D SPT trajectories formatted as time-series of x, y, (z), t coordinates	Interpretation of 2D and 3D SPT data extracting biological insights from motion	High segmentation accuracy, outperforms MDS- and LSTM-based methods by 10–30%	Allows to extract functional and localization information from motion alone. **Limitations**: complex set up, requires labelled training got custom task.	[108]
SEMORE	Multi-layered density-based clustering + morphology fingerprinting (40 + geometric/kinetic descriptors)	Raw SMLM data (x, y,t coordinates from STORM, PALM, DNA-PAINT, sptPALM); 4D time-resolved data	Heterogeneous protein aggregates (e.g., insulin spherulites); Nuclear pore complexes; Fibroblast growth receptors; Syntaxin 1a clusters	> 85% accuracy in simulated data classification > 90% accuracy in insulin aggregate pathway classification	System-agnostic analysis: handles overlapping structures & temporal evolution; Minimal user input required; Processes ~ 100k localizations in minutes. **Limitations**: Requires ≥ 10 localizations per structure; GPU needed for large 4D datasets (> 50 μm³ volumes)	[120]

## Clinical and personalized genomics applications for precision diagnostics and therapy

Recent advances in DL have revolutionized the extrapolation of cell morphology and chromatin structural features from images, providing new avenues for disease stratification, immune profiling, and therapeutic decision-making [24–28, 123–127]. A growing number of studies demonstrate that DL models can classify cell types, detect disease states, and predict treatment response using nuclear or chromatin organization as input (Table 4*).* The integration of imaging, genomics, and AI is redefining the limits of functional genomics and diagnostic innovation. In the following sections, we highlight key achievements and emerging applications that exemplify this convergence.

**Table 4:** Summary of AI approaches in nuclear and chromatin imaging

Study	Model Architecture	Imaging Input	Core Biological Application	Ref.
Sommer et al., (2017)	CellCognition Explorer: CNN autoencoder + novelty detection	Fluorescence images of nuclei (40 × 40 px)	Phenotype discovery in high-content screening	[124]
Yang et al., (2021)	Cross-modal autoencoders	DAPI-stained T-cell nuclei + scRNA-seq	Predict expression and cell subtype from chromatin image	[24]
Venkatachalapathy et al., (2021)	StarDist + ANN	Hoechst-stained nuclei in breast tissue	Pseudo-time progression modeling in breast cancer	[128]
Heinemann et al., (2022)	CNN (weak supervision)	20 × 20 px dual-channel confocal (DAPI, BF)	Drug response prediction in hematologic malignancies	[123]
Severin et al., (2022)	Custom CNN	5-channel immunofluorescence of PBMCs	Immune cell phenotyping and health profiling	[25]
Zhang et al., (2022)	CNN + Graph-based Autoencoder (STACI)	DAPI stained nuclei + STARmap transcriptomic data	Morphology-informed spatial transcriptomics in Alzheimer’s	[126]
Challa et al., (2023)	Random Forest (hand-crafted features)	3D confocal PBMCs (DAPI, γH2AX, Lamin A/C)	Liquid biopsy biomarkers and treatment monitoring	[125]
Daneshkhah et al., (2023)	VGG16 model	csPWS microscopy of buccal cell nuclei	Early-stage lung cancer detection in field carcinogenesis of buccal epithelium	[29]
Severin et al., (2024)	ResNet (71-layer CNN)	5-channels multiplexed AML bone marrow images	Classification of AML differentiation hierarchy and drug response	[26]
Carnevali et al., (2024)	DenseNet-121	Dual-color STORM (H3, Pol II)	Classification of pluripotent, somatic, and infected cells	[129]
Soelistyo & Lowe (2024)	β-TCVAE + Sparse NN + Symbolic Regression	Chromatin-stained fluorescence MDCK cells	Transparent classification of mitosis stages	[27]
Zhang et al., (2024)	Variational Autoencoder	DAPI-stained breast tissue nuclei	Spatial mapping of chromatin heterogeneity in DCIS	[127]

Among earlier contributions, Sommer and colleagues introduced CellCognition Explorer, a DL framework combining autoencoders and novelty detection for high-content image-based screening [124]. CellCognition Explorer enables phenotype discovery from nuclear morphology in large-scale RNAi screens without requiring manual training data. Applied to mitotic progression and nuclear shape classification tasks, CellCognition Explorer demonstrated the utility of unsupervised learning for rare or unexpected phenotype identification in functional genomics, marking an early success in the integration of DL and image-based screening.

The potential of DL to reveal subtle, disease-relevant nuclear phenotypes has been further demonstrated by Venkatachalapathy [28], who combined StarDist-based segmentation with ANN classifiers trained on Hoechst-stained images to classify tumor progression stages in breast cancer by encoding chromatin morphology into a pseudo-time score. Similarly, Heinemann and colleagues [123] introduced a weakly supervised CNN framework for predicting ex vivo drug response. This approach, termed deep morphology learning (DML), was trained on compact dual-channel confocal microscopy images (DAPI and brightfield, 20 × 20 pixels) to distinguish malignant from healthy cells based solely on nuclear morphology. This morphology-guided model improved stratification and reproducibility in functional drug screening, offering an alternative to molecular biomarker-based prediction.

Expanding into immunology, Severin et al., (2022) applied a custom DL classifier to multiplexed immunofluorescence images of peripheral blood mononuclear cells (PBMCs) [25]. The model, trained on five-channels input (four fluorescence plus brightfield), distinguished eight immune cell subtypes and revealed morphometric signatures that correlated with donor-specific health indicators such as systemic inflammation and age. This approach, referred to as deep immune phenotyping, highlights the potential of DL to non-invasively map immune diversity and systemic health. Building on this, Severin et al., (2024) trained a 71-layer ResNet model to stratify immunofluorescence images of bone marrow cells in acute myeloid leukemia (AML) [26], enabling the prediction of drug responses at the level of specific AML subpopulations, facilitating a personalized approach to treatment.

Alongside treatment prediction, DL-powered chromatin imaging has allowed the early diagnosis of various diseases. Challa et al., (2023) developed an AI-powered imaging pipeline to profile 3D chromatin organization in PBMCs from pan-tumor patients undergoing radiation therapy, using morphological features as biomarkers for both diagnosis and longitudinal treatment response [125]. Similarly, Daneshkhah et al., (2023) developed chromatin-sensitive partial wave spectroscopic microscopy (csPWS), an optical nanosensing method to non-invasively detect early-stage lung cancer [29]. This technique targets chromatin structural changes indicative of field carcinogenesis, a phenomenon wherein cells distant from the tumor site exhibit molecular signatures of cancer. By applying AI to csPWS data, the authors enhanced diagnostic accuracy: their AI-enhanced nanocytology distinguished Stage-I lung cancer from controls with AUCs of 0.92 and 0.82 across two clinical sites. Moreover, an effective tumor biomarker for invasive breast cancer was studied by Zhang et al., (2024), which used a convolutional VAE to analyze DAPI-stained nuclei in breast tissue microarrays [127]. Tissue-level analysis reveals that changes in the spatial organization of cell states across disease stages can effectively predict both disease progression and phenotypic category, establishing chromatin imaging as a powerful and accessible biomarker.

Still focusing on disease diagnostics, the integration of molecular readouts with imaging represents another key advancement. In the field of neuroscience, Zhang et al., (2022) introduced STACI, a graph-based multimodal autoencoder that fuses spatial transcriptomics data and DAPI images. Applied to mouse brain tissue, the model aligned chromatin morphology with spatial gene expression, enabling identification of morpho-molecular biomarkers in Alzheimer’s disease [126]. On the other hand, Yang et al., (2021) developed a cross-modal autoencoder architecture to embed chromatin images and RNA-seq profiles into a shared latent space [24]. Using matched DAPI-stained images and scRNA-seq data from naïve T cells, the model predicted transcriptional activity from nuclear morphology and identifies active T-cell sup-populations, highlighting the coupling of structural and functional cell states.

At the subnuclear level, Carnevali et al., (2024) presented AINU (Artificial Intelligence of the Nucleus), a CNN-based model trained on super-resolution STORM images of nuclear proteins, including histone H3 and RNA polymerase II [129]. Leveraging transfer learning with a DenseNet-121 backbone and retraining it with a small amount of cell images, AINU achieved high classification accuracy in distinguishing pluripotent, somatic, and virally infected human cells, demonstrating how nanoscale chromatin organization patterns can be harnessed to infer cellular states. Moreover, interpretable AI identified a specific nucleolar structure critical for classifying pluripotent cells, enabling the understanding of key biological features [129]. Similarly, in the context of interpretability and feature disentanglement, Soelistyo and Lowe (2024) proposed a framework that integrates a variant of the VAE model with symbolic regression and sparsity constraints [27]. Trained on fluorescence images of chromatin-stained cells, their method generated compact symbolic expressions that classified cell-cycle states, offering transparency in the mapping between morphology and function.

Together, these studies illustrate how deep-learning models operating across imaging scales, from tissue-level nuclear morphology down to nanometer-resolution chromatin, can support precise, interpretable cellular phenotyping. Furthermore, these advances are making chromatin analysis using single-molecule localization microscopy (SMLM) more efficient, demonstrating significant potential for clinical imaging. As the field advances, these developments are paving the way for non-invasive diagnostics, personalized therapies, and integrative spatial omics. Looking ahead, the integration of super-resolution microscopy with AI models could transform genomic medicine by enabling highly sensitive, non-invasive diagnostics and guiding targeted therapeutic interventions informed by subnuclear chromatin architecture.

## Challenges and limitations

Despite the transformative potential of AI in chromatin and nuclear architecture research, several key challenges remain.

### Ensuring trustworthy models: benchmarking, interpretability, and biological validation

High-resolution chromatin imaging and genomic assays are often limited by low throughput, phototoxicity, and experimental variability. Training robust DL models requires large, well-annotated datasets, which are still scarce in many biological domains. While recent efforts have produced specialized datasets like **BioTISR** [130] (collection of paired low/high-resolution 2D and 3D time-lapse biological images for super-resolution microscopy) and **SR-CACO-2** [131] (9,937 real low/high resolution patches for confocal fluorescence microscopy), these remain exceptions rather than norms. The **DL-SMLM** dataset further exemplifies progress, providing 188 raw SMLM datasets with six subcellular structures and 100 signal levels per image to support DL training [132]. However, most biological domains still lack large, well-annotated datasets, forcing researchers to rely on synthetic data that often fail to capture sample-specific aberrations [133]. Moreover, differences in imaging platforms, staining protocols, and sample preparation further introduce batch effects that complicate model generalization. The **MICCAI Federated Tumor Segmentation Initiative** demonstrated that aggregating model updates from numerous sites improved generalizability [134], but such collaborative frameworks remain underutilized in chromatin imaging. Publication of more labelled datasets and unifying evaluation techniques could help in overcoming the technical challenge of generalization models across different domains.

Currently, there is no standardized benchmarking dataset or evaluation metric tailored specifically for chromatin-focused AI models. This lack of common ground hampers fair comparison across methods and ultimately slows scientific progress. Moreover, open access to training datasets, model weights, and evaluation scripts is essential to foster reproducibility but is not yet a common practice.

Furthermore, DL models are often criticized for their “black-box” nature. Saliency mapping, which identifies input regions most influential to model predictions, has been widely adopted in microscopy [135]. However, studies reveal that saliency maps often lack robustness—for example, models trained on medical imaging datasets showed inconsistent sensitivity to weight randomization, with many highlighted regions failing to correspond to meaningful biological structures [135, 136]. Region-based interpretability methods, such as Gradient-weighted Class Activation Mapping (Grad-CAM) and occlusion analysis, are commonly used in natural image interpretation. Yet, their application in biological domains remains limited due to the need for domain-specific validation and expert interpretation [129], what makes translating these visualizations into biologically actionable hypotheses remains challenging [128, 129]. Without rigorous benchmarking and orthogonal experimental validation, there is a substantial risk of overinterpreting AI-derived outputs and drawing biologically incorrect conclusions.

### Making AI accessible for biological research

The integration of artificial intelligence in biological research has reached a critical juncture where technological capability increasingly outpaces accessibility for the broader research community. While deep learning methods have demonstrated remarkable potential to revolutionize microscopy image analysis, segmentation, and biological discovery, their adoption remains a major barrier in advancing biological discovery, particularly for researchers without a background in programming or data science. This challenge is reflected in the relatively low number of publications employing these methods to investigate biological questions in super resolution imaging [137].

To fully harness the potential of AI in chromatin studies, there is a pressing need to develop more accessible tools and foster interdisciplinary collaboration between computational experts, biologists and clinicians. The complexity extends beyond mere technical implementation to encompass fundamental issues of computational infrastructure and resource allocation. Deep learning applications in biological imaging often require substantial GPU computing power, particularly for tasks involving super-resolution reconstruction, 3D modeling, and high-fidelity dynamics tracking. These computational demands frequently create a digital divide, concentrating analytical power in well-funded institutions and limiting broader adoption. Further, the lack of standardized workflows and user-friendly interfaces compounds these difficulties, often requiring researchers to navigate complex documentation and troubleshoot technical issues that fall outside their primary areas of expertise.

Recent developments in user-friendly, cloud-based, and GUI-driven platforms tend to overcome these challenges, enabling researchers without computational expertise to apply advanced AI methods to 3D genome imaging. ZeroCostDL4Mic is an example of entry-level platform that allows researchers with zero computational expertise to train and apply DL models for different tasks [138]. This platform, leveraging Google Collab resources, includes segmentation, object detection, SMLM image reconstruction and denoising tools, allowing for their easy application in research. Beyond basic functionality, ZeroCostDL4Mic incorporates sophisticated evaluation frameworks that ensure model reliability through comprehensive quality control metrics. Additionally, trained models can be exported for use with other established platforms such as ImageJ2, Fiji, and specialized analysis software, ensuring compatibility with existing research workflows [139, 140]. Similarly, SEMORE offers an end-to-end, semi-automatic framework for analyzing super-resolution microscopy data with temporal and morphological analysis, enabling detailed study of dynamic biological processes without extensive computational knowledge [120]. The napari ecosystem has emerged as a powerful platform for democratizing access to advanced image analysis through its extensible plugin architecture and provides accessible interfaces for segmentation, annotation, and model fine-tuning, supporting diverse imaging modalities and workflows [141]. Tools like napari-nucleaizer [142] facilitate accurate nuclear segmentation across modalities, while napari-activelearning and napari-ndev support iterative model tuning and batch processing for high-content imaging. These tools are particularly relevant for chromatin studies that require precise nucleus delineation, volumetric analysis, or temporal tracking. For integration with traditional workflows, DeepImageJ and Ilastik [143] provide accessible solutions within the familiar environments of ImageJ and interactive annotation tools, supporting nuclear classification and segmentation without coding. Meanwhile, DeepCell Kiosk [144] offers scalable, cloud-native processing for large datasets—ideal for high-throughput chromatin imaging.

Collectively, these platforms bridge the gap between innovation and biological relevance, enabling broader exploration of chromatin structure, nuclear organization, and genome architecture at scale. Allowing easy access to these and future deep learning approaches is essential for the improvement of research and its clinical application.

## Conclusions and future outlook

The advent of ML models has greatly helped to reveal many aspects of biology. Within these, the correlation between chromatin folding and gene expression, epigenetic states, and cellular identity, unlocking a level of mechanistic insight that was previously unattainable. Indeed, the fusion of AI with high-resolution imaging and multi-omics is redefining our understanding of chromatin architecture and nuclear function. More precisely, AI-driven tools have enabled accurate segmentation, classification, 3D modeling, and dynamic analysis of nuclear structures at nanoscale resolution.

Beyond descriptive modeling, AI is beginning to guide hypothesis generation and experimental design. Saliency mapping, attention mechanisms, and interpretable architectures are shedding light on which nuclear features drive gene regulatory decisions. As the imaging and analysis methods in the field of 3D genome organization improve, temporal modeling and live-cell tracking are offering new perspectives on genome behavior in development, differentiation, and disease. As examples, recent publications such as Gabriele et al., (2022), Mach et al., (2022) and Mazzocca et al., (2025) present transformative approaches to investigating chromatin loop dynamics [145–147]. This collection of groundbreaking studies marks a transformative leap in our understanding of chromatin loop dynamics, moving towards a dynamic, time-resolved view of genome architecture in living cells. Combination with novel SR imaging techniques in living cells, such as MINFLUX and super-resolution live-cell imaging (SRLCI), with rigorous statistical modeling, genetic engineering, and polymer simulations, they revealed the rare, dynamic and short-lived nature of CTCF- and cohesin-mediated chromatin loops. Such findings challenge conventional assumptions about TADs and highlight the stochastic, subdiffusive nature of chromatin motion, with great implications for processes like enhancer-promoter communication and DNA repair. Looking ahead, integration of AI on these novel datasets could greatly improve the prediction of loop formation and the modelling of chromatin motion.

Looking forward, several frontiers remain. The integration of real-time multi-modal data, combining chromatin imaging, RNA dynamics, proteomics, and mechanobiology, will require new classes of AI architectures capable of reasoning across time and space scales. The democratization of AI tools, via open-source frameworks, cloud platforms, and standardized benchmarks, will be critical to ensure accessibility and reproducibility.

In clinical contexts, AI-enhanced chromatin analysis is emerging as a non-invasive diagnostic modality, offering early detection and personalized treatment insights. Ethical guidelines, transparency standards, and collaborative validation will be essential to ensure safe deployment in biomedical settings.

For AI to be more widely acceptable as a clinical decision support tool, it should strive in explainability. A user should be able to understand the reasoning behind a model prediction. This way, a specialist will be able to interpret the outcome of a model correlating the findings with the medical conditions and data of the patient. Exploring the connection between machine-generated findings and patients’ medical conditions and leveraging DL and big data analytics to identify new associations between diseases and clinical data or symptoms, will be a critical research focus. This will help Computer-Aided Diagnosis (CAD) provide interpretable results to clinicians and drive the evolution of CAD toward true AI in medicine.

In conclusion, AI is not merely a tool but a transformative framework in chromatin biology. As algorithms grow more interpretable, multimodal, and generalizable, they will become indispensable partners in decoding the spatial and functional logic of the genome. This convergence of AI and nuclear science stands to illuminate not just how genes are regulated, but how life is organized at its most fundamental level.

## Data Availability

Not applicable.
